# Visceral Adiposity as an Independent Risk Factor for Diabetic Peripheral Neuropathy in Type 2 Diabetes Mellitus: A Retrospective Study

**DOI:** 10.1155/2024/9912907

**Published:** 2024-11-11

**Authors:** Rui-Ling Wu, Niyao Chen, Yanni Chen, Xiaohong Wu, Chih-Yuan Ko, Xiao-Yu Chen

**Affiliations:** ^1^Department of Clinical Nutrition, The Second Affiliated Hospital of Fujian Medical University, Quanzhou, China; ^2^Department of Endocrinology and Metabolism, The Second Affiliated Hospital of Fujian Medical University, Quanzhou, China; ^3^Department of Endocrinology, Shishi General Hospital, Quanzhou, China

**Keywords:** bioelectrical impedance analysis, body composition analysis, diabetes peripheral neuropathy, obesity, Type 2 diabetes mellitus, visceral fat

## Abstract

**Background:** Diabetic peripheral neuropathy (DPN) impacts approximately 50% of individuals with Type 2 diabetes mellitus (T2DM), leading to severe complications such as foot ulcers and amputations. Notably, visceral adiposity is increasingly recognized as a pivotal factor in augmenting the risk of DPN. We aim to evaluate the correlation between obesity-related body composition, particularly visceral fat, and DPN to facilitate early identification of high-risk patients with T2DM.

**Methods:** This cross-sectional analysis encompassed 113 T2DM patients from the Department of Endocrinology and Metabolism at the Second Affiliated Hospital of Fujian Medical University, conducted between September 2020 and January 2021. Patients were categorized into two cohorts: those with DPN (DPN group) and those without (NDPN group). We utilized bioelectrical impedance analysis (BIA) to determine body measurements, such as weight and visceral fat area, in addition to collecting clinical and biochemical data. Logistic regression was employed to analyze the data.

**Results:** The study uncovered a statistically significant difference in the visceral fat area between the DPN and NDPN groups (*p* = 0.048). Through multivariate logistic regression analysis, the visceral fat area was identified as an independent risk factor for DPN among T2DM patients (OR 1.027; 95% CI 1.004–1.051, *p* = 0.022). Other significant risk factors included the duration of diabetes and the presence of diabetic retinopathy.

**Conclusion:** The visceral fat area serves as an independent risk factor for DPN in individuals with T2DM. Implementing measures to assess and manage visceral obesity could be vital in the prevention and management of DPN. This underscores the value of technologies such as BIA in clinical and community settings for early intervention.

## 1. Introduction

Globally, diabetes has become a significant public health concern, with its prevalence increasing annually. A cross-sectional survey of 98,658 Chinese adults conducted in 2010 revealed a diabetes prevalence rate of 11.6% [1]. Diabetes peripheral neuropathy (DPN), one of the most common chronic complications during the progression of Type 2 diabetes mellitus (T2DM), affects 25%–50% of individuals with the condition [2, 3]. It has been shown that DPN increases the risk of falls, foot ulcers, and lower limb amputations [2, 3]. The early detection of DPN is beneficial in slowing the disease's progression and reducing foot ulcer incidence and mortality [4]. The pathogenesis of DPN is complex, involving various factors such as inflammation, oxidative stress, mitochondrial dysfunction, and alterations in gene expression [5]. The underlying nerve damage in DPN cannot be effectively treated at present. For the management of DPN, prevention remains the most effective strategy. In order to better understand the pathogenesis of DPN and develop effective prevention strategies, it is essential to gain a better understanding of its risk factors.

It is not uncommon for individuals with T2DM to have a higher body weight. In addition, it increases the health risks associated with T2DM and complicates its management [6]. It is not uncommon for individuals with T2DM to have a higher body weight [7–9]. Higher body weight is a significant contributor to the prevalence of DPN as it is associated with metabolic syndrome [10]. There are a variety of body locations where obesity can manifest, including subcutaneous and visceral depots, as well as nonadipose tissues (ectopic fat deposits) [11]. In regard to body fat distribution, obesity can be broadly categorized into central obesity, which predominantly involves accumulation of visceral or deep subcutaneous fat, and general obesity, which is characterized by a relatively more widespread distribution of fat. However, it is crucial to note that individuals with general obesity exhibit considerable variability in the extent and proportion of visceral to subcutaneous fat. This variability underscores the complexity of obesity-related metabolic diseases, which are influenced not only by total fat mass but also by the specific distribution and functional characteristics of adipose tissue. Such distinctions are critical as they may differentially contribute to the risk and development of obesity-related complications [12]. A strong association has been found between central obesity and certain diabetes complications [13]. Individuals with central obesity, particularly those with excess visceral fat, are at increased risk of metabolic and cardiovascular diseases [14]. Metabolic diseases related to higher body weight are closely associated with visceral fat. Individuals with minimal visceral fat and severe obesity may be more insulin sensitive. However, those with more visceral adipose tissue may exhibit insulin resistance and dyslipidemia and be more likely to develop T2DM and cardiovascular disease [15]. A recent study demonstrated that preperitoneal fat (a component of visceral fat) negatively regulates diabetic neuropathy through ultrasonography [16]. The area of visceral fat in T2DM patients is also associated with a decline in cardiovascular autonomic function and insulin resistance [17]. It is therefore possible that central obesity, in particular visceral adipose obesity, has a more intimate connection to DPN.

As obesity has progressed beyond traditional measurements such as body mass index (BMI), waist circumference, hip circumference, and waist-to-hip ratio, body composition analysis has gained traction in the measurement of human fat. To assess fat distribution, particularly visceral fat, current methods include computed tomography (CT), magnetic resonance imaging (MRI), ultrasound, dual-energy X-ray absorption (DXA), and bioelectrical impedance analysis (BIA). As the gold standard for measuring visceral fat, CT is limited by concerns regarding radiation exposure, high costs, and the complexity of equipment and procedures. Due to its high cost, lengthy scan durations, and requirement for considerable cooperation from the patient, MRI is less frequently utilized when attempting to quantify visceral fat. A lack of uniform measurement markers and the need for precise positioning and probe pressure make ultrasound measurement difficult to repeat [18]. While DXA is advantageous in measuring total body fat content, it is insufficient for assessing longitudinal changes in visceral fat and is associated with low-dose radiation exposure [18]. BIA, which measures resistance by passing a weak current through the body, assessing fat content and distribution, offers the simplicity of anthropometric measurements. It has been demonstrated that BIA is more reliable than CT [19], making it useful for the assessment of the content and distribution of body fat, with particular advantages in the measurement of visceral fat.

There is, however, a paucity of research using body composition analysis, including BIA, to investigate the relationship between obesity, particularly visceral adiposity, and DPN. This study is aimed at using BIA, a body composition analyzer, at quantifying obesity-related indicators, particularly visceral fat area (VFA), and at determining the relationship between the VFA and DPN in individuals with T2DM.

## 2. Materials and Methods

### 2.1. Participants

This study was a single-center cross-sectional retrospective study. The institutional ethics committee (Ethics Review Number 2021-31) approved all procedures. Diabetes was diagnosed according to the WHO criteria proposed in 1999 [20]. All patients with T2DM were recruited from the Department of Endocrinology and Metabolism, the Second Affiliated Hospital of Fujian Medical University, from September 2020 to January 2021. The inclusion criteria specified patients with a definitive diagnosis of T2DM. There were several exclusion criteria, including individuals younger than 18 years old or older than 80 years old, those with other types of diabetes, those with acute complications of diabetes, and those with infections or tumors. The study was also exclusionary for individuals with severe liver function abnormalities (transaminase levels threefold above the upper normal limit), end-stage renal disease, heart failure, edema, muscle atrophy, dehydration, and oral diuretics, as well as those with conditions such as edema, muscle atrophy, and dehydration and oral diuretic therapy. Furthermore, individuals with cardiac stents, pacemakers, or any other metal implants were excluded from participation.

### 2.2. DPN Diagnosis

Based on the diagnostic criteria established by the Chinese Medical Association's Diabetes Society [21], participants were categorized according to their presence of DPN. As a result, the study differentiated between T2DM patients with DPN (DPN group) and those without DPN (NDPN group). This diagnosis was based on a clear diabetes history, the development of neuropathy at the time of diabetes diagnosis or afterwards, and the presence of clinical symptoms and signs characteristic of DPN. The diagnosis of symptomatic individuals required at least one abnormal result across five examinations (ankle reflexes, pinprick, vibration, pressure, and temperature sensation). Asymptomatic individuals require at least two abnormalities for a DPN diagnosis. As part of the exclusion list for DPN diagnosis, neuropathies from other causes, such as cervical or lumbar spine disease, cerebral infarction, Guillain–Barre syndrome, serious arteriovenous vascular diseases (including venous thrombosis and lymphangitis), and metabolic toxins resulting from renal insufficiency–affected nerves, were included. Diagnosis of DPN was classified as confirmed (presence of symptoms/signs of distal symmetrical polyneuropathy in diabetes with abnormal nerve conduction), clinical diagnosis (symptoms and one positive sign, or no symptoms with two or more positive signs), suspected (symptoms without signs, or no symptoms with one positive sign), and subclinical (no symptoms/signs, only abnormal nerve conduction). DPN cases in the T2DM group had been confirmed in the DPN group as well.

### 2.3. Body Composition Measurement

Body composition measurements were conducted using the multifrequency tetrapolar InBody S10 device (Biospace Co, Ltd., Seoul, Korea), following the procedures outlined in our previous studies [22, 23].

### 2.4. Eye Examination

Ophthalmologists performed the examination. For fundoscopic examination, a Topcon SL-3G slit lamp was used with a VOLK +90 D lens, followed by further investigation with a Canon CR-2 retinal camera. To assess the severity of retinal lesions, the retinal camera findings were compared to those of the lens examination. The macular region of the retina was scanned by spectral domain optical coherence tomography (Heidelberg Spectralis SD-OCT). Ophthalmologists assessed and diagnosed diabetic retinopathy based on the collective findings.

### 2.5. Blood Biochemistry Analysis

We obtained 4 mL of venous fasting blood from each participant. We conducted biochemical assessment using the Mindray BS-280 Fully Automated Chemical Analyzer (NMT, Shenzhen, China), which measured a number of parameters, including alanine aminotransferase (ALT), aspartate aminotransferase (AST), total cholesterol (TC), triglycerides (TGs), fasting plasma glucose (FPG), uric acid (UA), serum creatinine (SCR), high-density lipoprotein (HDL), and low-density lipoprotein (LDL). It was estimated that the estimated glomerular filtration rate (eGFR) was equal to 186 × (SCR [*μ*mol/L]/88.402)^−1.154^ × age^−0.203^ × (female × 0.742) [24].

### 2.6. Glycated Hemoglobin (HbA1c) and Fasting C-Peptide (FC) Measurements

We collected 2 mL of fasting venous blood from each participant for analysis. A high-performance liquid chromatography system from Bio-Rad Laboratories, United States, was used to measure the level of HbA1c. Chemiluminescence was used to measure FC levels using a fully automated immunoassay analyzer (Liaison XL, DiaSorin S.p.A, Vercelli, Italy).

### 2.7. Assessment of Urine Albumin-to-Creatinine Ratio (UACR)

The UACR was determined by collecting a morning midstream clean-catch urine sample from the patients. UACR was calculated following the measurement of urinary microalbumin and urinary creatinine with the colorimetric method using diacetyl monoxime.

### 2.8. Statistical Analysis

SPSS software Version 26.0 was used for statistical analysis. Using the Kolmogorov–Smirnov test, we verified the normality and homogeneity of the variances in the data. Quantitative data following a normal distribution were presented as mean ± standard deviation, whereas nonnormally distributed data were described as median (interquartile range). Normally distributed data were compared using the independent samples *t*-test, while nonnormally distributed data were compared using the Mann–Whitney *U* nonparametric test. Categorical data were expressed as frequencies and percentages, and intergroup comparisons were conducted using the chi-square test. Correlations between two continuously distributed variables following a normal distribution were analyzed using Pearson's correlation and Spearman's correlation, respectively. Univariate logistic regression analysis was employed to evaluate the association between DPN and its potential influencing factors in T2DM patients. The multivariate logistic regression analysis was adjusted for several variables including gender, duration of diabetes, FPG, HbA1c, UA, BMI, VFA, and diabetic retinopathy to assess their impact on the developing DPN. Statistical significance was determined by *p* values less than 0.05 in all tests.

## 3. Results

### 3.1. Clinical Characteristics and Blood Biochemistry

This study finally included 113 patients with T2DM, of whom 50 were in the DPN group and 63 in the NDPN group. The DPN group consisted of 34 males and 16 females, with an average age of 57.50 ± 11.13 years; the NDPN group comprised 33 males and 30 females, with an average age of 55.17 ± 10.76 years. There were no statistically significant differences between the two groups in gender and age (Table 1). Compared to patients in the NDPN group, those in the DPN group had a longer duration of diabetes (*p* = 0.001) and higher levels of FPG (*p* = 0.034), HbA1c (*p* = 0.023), and UA (*p* = 0.039). Additionally, the proportion of patients with diabetic retinopathy in the DPN group was significantly higher than that in the NDPN group (*p* < 0.001) (Table 1).

### 3.2. Body Composition of the DPN Group and NDPN Group

Among all participants, the VFA ranged from 22.6 to 210.6 cm^2^. In the DPN group, the VFA ranged from 24.1 to 210.6 cm^2^, whereas in the NDPN group, it ranged from 22.6 to 195.7 cm^2^. Compared to the NDPN group, patients in the DPN group had significantly higher body weight (*p* = 0.012), waist circumference (*p* = 0.015), and VFA (*p* = 0.048) (Table 2). However, there was no significant difference between the two groups in terms of BMI, total body fat mass, and percentage of body fat (Table 2).

### 3.3. Correlation Data

Several continuous variables were correlated using Pearson's or Spearman's correlation, including diabetes duration, FPG, HbA1c, UA, weight, waist circumference, and VFA. The results demonstrated a significant positive correlation between HbA1c and FPG (*r* = 0.618, *p* < 0.01) (Table 3), underscoring a direct relationship between these two critical markers of glycemic control. FPG and HbA1c represent distinct facets of glucose homeostasis. FPG measures immediate plasma glucose concentrations and is susceptible to short-term dietary fluctuations, while HbA1c offers a mean estimation of blood glucose levels over approximately 3 months, thus reflecting sustained glycemic control. Both parameters are utilized independently in clinical settings to monitor diabetes management and have consistently been linked to diabetic complications across numerous studies. FPG is typically employed for rapid adjustments in therapy, whereas HbA1c serves as a more reliable indicator of long-term complications and overall disease progression.

Additionally, the VFA shows a positive correlation with the duration of diabetes (*r* = 0.288, *p* = 0.002). Furthermore, body weight is positively correlated not only with the duration of diabetes (*r* = 0.213, *p* = 0.023) but also with levels of UA (*r* = 0.373, *p* < 0.01) and the extent of VFA (*r* = 0.621, *p* < 0.01). Similarly, waist circumference, an indicator of central adiposity, is positively correlated with the duration of diabetes (*r* = 0.275, *p* = 0.003), UA (*r* = 0.264, *p* = 0.005), VFA (*r* = 0.853, *p* < 0.01), and overall body weight (*r* = 0.893, *p* < 0.01) (Table 3).

### 3.4. Univariate Logistic Regression Analysis of DPN in Patients With T2DM

In a univariate logistic regression analysis, it was determined that the duration of diabetes (*p* = 0.001), FPG (*p* = 0.027), weight (*p* = 0.016), waist circumference (*p* = 0.019), VFA (*p* = 0.044), and diabetic retinopathy (*p* < 0.001) are significant risk factors for the development of DPN in Type 2 diabetes patients, with all associations showing a positive direction (Table 4).

### 3.5. Multivariate Logistic Regression Analysis of DPN in Patients With T2DM

For multivariate logistic regression analysis, after adjusting for variables such as gender, duration of diabetes, FPG, HbA1c, UA, BMI, VFA, and diabetic retinopathy, our analysis revealed that visceral fat is a significant independent risk factor for DPN in patients with T2DM. Specifically, each unit increase in the VFA was associated with a 2.7% increase in the risk of developing DPN (OR 1.027 [95% CI 1.004, 1.051], *p* = 0.022). Furthermore, the duration of diabetes was also identified as an independent risk factor for DPN (OR 1.009 [95% CI 1.002, 1.016], *p* = 0.007). Patients with diabetic retinopathy exhibited a significantly higher probability of having DPN, with odds 3.909 times greater than those without retinopathy (OR 3.909 [95% CI 1.373, 11.127], *p* = 0.011) (Table 5).

## 4. Discussion

In this study, we employed BIA to measure the VFA in patients with T2DM and explored its relationship to DPN. Traditionally, research on obesity and DPN has focused on anthropometric indices such as BMI, waist circumference, waist-to-hip ratio, and waist-to-height ratio. However, few studies have assessed visceral adiposity using BIA in conjunction with a body composition analyzer to determine its specific impact on DPN. Our findings suggest that visceral adiposity, as identified by BIA, plays a critical role in the early detection and management of DPN. The use of BIA offers a practical approach for diagnosing and treating at-risk individuals in community settings by accurately measuring visceral fat levels.

In our cohort, patients with T2DM and DPN exhibited higher body weight, waist circumference, and VFA compared to those without neuropathy. Notably, other metrics such as BMI, total body fat, and body fat percentage did not show significant differences, highlighting that DPN is more closely associated with the distribution of fat rather than the overall quantity. Supporting literature indicates that waist circumference, rather than BMI, is more closely linked to DPN [24, 25], with recent studies also emphasizing the significant relationship between central obesity and DPN [9], reinforcing the importance of focusing on visceral rather than peripheral fat.

Our analysis also indicates that waist circumference is positively correlated with VFA in T2DM patients, confirming that increases in waist circumference may be indicative of increased visceral fat [26]. Among patients with both T2DM and DPN, visceral obesity is the predominant form of central obesity. Although it has been proposed that waist circumference alone could serve as a sufficient marker of visceral fat [27], our results suggest that direct assessment of visceral fat content is necessary to fully understand the implications of increased waist circumference.

Furthermore, visceral and subcutaneous adiposities, the two primary forms of central obesity, appear to differ significantly in their associations with health risks [28]. Visceral adiposity, often termed “dangerous obesity,” is more strongly correlated with negative outcomes than subcutaneous obesity [12]. This is likely due to its association with insulin resistance and adipose tissue dysfunction, which can lead to a metabolic inflammatory response, further exacerbating metabolic diseases.

One important connection between central obesity, especially visceral obesity, and higher disease risk may be insulin resistance [29]. Adipose tissue dysfunction can also result from increased visceral depot fat storage. This can cause a low-grade metabolic inflammatory response, which in turn can promote metabolic diseases [12]. Metabolic inflammation in patients with T2DM is likely mediated primarily by proinflammatory adipokines secreted by visceral and ectopic adipocytes, resulting in damage to the peripheral nerves and microvascular system [30]. As a result of altered secretion and distribution of adipokines, impaired insulin signaling, disrupted TG storage, and increased basal lipolysis, elevated levels of circulating adipokines and free fatty acids are produced, resulting in peripheral tissue and nerve dysfunction [28]. When free fatty acids are elevated excessively, they cause inflammation in sensory neurons and oxidative stress, leading to endoplasmic reticulum stress, mitochondrial dysfunction, cellular damage, and irreversible damage [3]. Using proinflammatory cytokines to predict distal sensorimotor polyneuropathy progression, it was found that certain subclinical inflammatory biomarkers were associated with the progress of the disease. Furthermore, it was confirmed that two chemokines, monocyte chemoattractant protein-3 and interferon-gamma inducible protein-10, as well as the neuron-specific marker Delta/Notch-like epidermal growth factor-related receptor, were potential mediators of metabolic inflammation resulting in DPN [27, 31].

Collectively, research has solidified the link between adiposity and DPN. Total body adiposity, hypertriglyceridemia, and leg fat have been identified as independent risk factors for DPN in Chinese Type 2 diabetes patients, potentially exacerbating sensory nerve damage through mechanisms such as inflammation and oxidative stress [11]. Similarly, a study has found comparable neuropathy risks in individuals with long-standing Type 1 diabetes linked to obesity, where elevated BMI and poor lipid profiles contribute to nerve dysfunction via insulin resistance and systemic inflammation [32]. These insights demonstrate that visceral adiposity impacts neuropathy through both mechanical stress and metabolic disruptions, highlighting the critical need for targeted weight and metabolic health management to mitigate neuropathy risks in diabetic populations.

Additionally, our study reveals the duration of diabetes as a risk factor for DPN, consistent with previous finding [10]. In our results, the prevalence of diabetic retinopathy in the DPN group was significantly higher than that in the NDPN group. Multivariate logistic regression analysis indicated that diabetic retinopathy is a risk factor for DPN among T2DM patients. A study has shown that the risk of developing diabetic retinopathy is increased in patients with DPN compared to those without DPN [33], likely due to shared pathological foundations related to glucose metabolism disorder, microvascular complications, and microcirculatory disturbances [34].

It is notable that low UA levels are inversely associated with DPN in patients with T2DM, especially affecting the motor conduction velocity of the tibial nerve. This observation suggests that reduced UA may itself be a risk factor for DPN [35]. Our study not only primarily investigates visceral adiposity as an independent risk factor for DPN but also illuminates the broader role of both UA and visceral adiposity. These factors are linked to oxidative stress and inflammation, which are central to the pathogenesis of DPN. UA acts as a natural antioxidant, potentially mitigating oxidative damage, while visceral adiposity fosters a proinflammatory state that may exacerbate nerve damage. Further supporting this, elevated UA levels have been correlated with various diabetic complications like nephropathy and retinopathy, which themselves are risk factors for DPN [36, 37]. Similarly, visceral adiposity is strongly connected to metabolic syndrome components and cardiovascular risk factors, underscoring the complex interplay of these elements in diabetes complications [14, 15].

Our study is constrained by several limitations. The cohort size is relatively small, and data collection was confined to a single center, which may restrict the generalizability of our findings and hinder the establishment of a definitive causal relationship between VFA and DPN. To elucidate these relationships further, prospective or interventional studies are warranted. Additionally, this study did not account for factors such as chronic alcohol consumption, cigarette smoking, and secondhand smoke exposure, which are known to affect hyperglycemia and DPN via mechanisms like enhanced insulin resistance and inflammatory responses [38]. These factors should be considered in future research endeavors. Moreover, no significant differences in age, gender, smoking habits, hypertension, and dyslipidemia were observed between the DPN and NDPN groups, which may be attributed to the limited sample size and the relatively low prevalence of peripheral neuropathy among the participants. We plan to continue data collection to increase the sample size and enable more comprehensive future analyses.

## 5. Conclusions

This study demonstrates VFA as a significant independent risk factor for DPN in T2DM patients. Findings underscore the necessity of early intervention and continuous monitoring using BIA to manage visceral obesity effectively. Additionally, prolonged diabetes duration and diabetic retinopathy are critical predictors of DPN, emphasizing the need for personalized, comprehensive management strategies focusing on metabolic health to prevent severe complications.

## Figures and Tables

**Table 1:** Clinical characteristics and blood biochemical parameters in the diabetic peripheral neuropathy (DPN) and nondiabetic peripheral neuropathy (NDPN) groups.

	**DPN group (** **N** = 50**)**	**NDPN group (** **N** = 63**)**	**p** ** value**
Male/female (*N*)	34/16	33/30	0.093
Age (years)	57.50 ± 11.13	55.17 ± 10.76	0.263
Smoking history (%)	20	23.8	0.628
Family history of diabetes (%)	28	39.7	0.194
Duration of diabetes (months)	114 (48, 159)	48 (6, 96)	**0.001**
History of hypertension (%)	38	31.7	0.487
SBP (mmHg)	133.58 ± 17.73	127.94 ± 14.16	0.063
DBP (mmHg)	81.00 (75.75, 87.75)	79.00 (75.00, 86.00)	0.428
FPG (mmol/L)	8.15 (6.33, 10.72)	6.72 (6.21, 8.06)	**0.034**
HbA1c (%)	8.80 (6.73, 10.70)	7.10 (6.30, 9.10)	**0.023**
FC (ng/mL)	1.86 (1.48, 2.94)	2.19 (1.56, 2.80)	0.799
TG (mmol/L)	1.47 (0.93, 2.20)	1.53 (1.02, 1.89)	0.926
TC (mmol/L)	4.30 (3.80, 5.21)	4.46 (3.80, 5.33)	0.521
LDL (mmol/L)	2.74 (1.88, 3.34)	2.73 (2.23, 3.68)	0.372
HDL (mmol/L)	1.12 (0.86, 1.45)	1.16 (1.00, 1.30)	0.931
ALT (U/L)	20.50 (13.75, 28.25)	19.00 (14.00, 32.00)	0.947
AST (U/L)	17.00 (14.00, 21.50)	19.00 (15.00, 23.00)	0.165
UA (*μ*mol/L)	337.50 (270.75, 384.25)	300.00 (251.00, 353.00)	**0.039**
SCR (*μ*mol/L)	68.00 (54.25, 84.00)	63.00 (53.00, 72.00)	0.242
UACR (mg/g)	22.13 (11.37, 44.28)	14.60 (9.24, 28.28)	0.05
eGFR (mL/min·1.73 m^2^)	105.26 ± 38.50	112.12 ± 35.17	0.326
Diabetic microvascular complications			
DR (%)	46	14.3	**< 0.001**
DKD (%)	38	22.2	0.067

*Note:* Data are presented as mean ± standard deviation, median (interquartile range), or percentages. Values in bold indicate that < 0.05 is considered to possess statistical significance.

Abbreviations: ALT, alanine aminotransferase; AST, aspartate aminotransferase; DBP, diastolic blood pressure; DKD, diabetic kidney disease; DR, diabetic retinopathy; eGFR, estimated glomerular filtration rate; FC, fasting C-peptide; FPG, fasting plasma glucose; HbA1c, glycated hemoglobin; HDL, high-density lipoprotein; LDL, low-density lipoprotein; SBP, systolic blood pressure; SCR, serum creatinine; TC, total cholesterol; TGs, triglycerides; UA, uric acid; UACR, urine albumin-to-creatinine ratio.

**Table 2:** Body composition in the diabetic peripheral neuropathy (DPN) and nondiabetic peripheral neuropathy (NDPN) groups.

	**DPN group (** **N** = 50**)**	**NDPN group (** **N** = 63**)**	**p** ** value**
Weight (kg)	70.26 ± 12.40	64.52 ± 11.53	**0.012**
Body mass index (BMI) (kg/m^2^)	25.10 ± 3.76	24.21 ± 3.24	0.178
Waist circumference (cm)	89.20 ± 11.69	84.31 ± 9.46	**0.015**
Body fat mass (kg)	20.81 ± 7.64	18.52 ± 6.70	0.093
Percentage of body fat (%)	29.10 ± 8.02	28.34 ± 7.86	0.611
Visceral fat area (cm^2^)	95.10 (65.03, 134.05)	82.80 (64.40, 99.80)	**0.048**

*Note:* Values in bold indicate that < 0.05 is considered to possess statistical significance.

**Table 3:** Correlation analysis of risk factors for diabetic peripheral neuropathy in patients with Type 2 diabetes mellitus.

	**Duration of diabetes**	**Fasting plasma glucose**	**Glycated hemoglobin**	**Uric acid**	**Visceral fat area**	**Weight**	**Waist circumference**
Duration of diabetes							
Fasting plasma glucose	*r* = 0.018 *p* = 0.854						
Glycated hemoglobin	*r* = −0.107 *p* = 0.258	**r** = 0.618 **p** < 0.01					
Uric acid	*r* = 0.058 *p* = 0.541	*r* = 0.073 *p* = 0.443	*r* = −0.085 *p* = 0.369				
Visceral fat area	**r** = 0.288 **p** = 0.002	*r* = −0.058 *p* = 0.539	*r* = −0.168 *p* = 0.076	*r* = 0.161 *p* = 0.088			
Weight	**r** = 0.213 **p** = 0.023	*r* = 0.083 *p* = 0.380	*r* = −0.041 *p* = 0.666	**r** = 0.373 **p** < 0.01	**r** = 0.621 **p** < 0.01		
Waist circumference	**r** = 0.275 **p** = 0.003	*r* = 0.027 *p* = 0.773	*r* = −0.087 *p* = 0.357	**r** = 0.264 **p** = 0.005	**r** = 0.853 **p** < 0.01	**r** = 0.893 **p** < 0.01	

*Note:* Values in bold indicate that < 0.05 is considered to possess statistical significance.

**Table 4:** Univariate logistic regression analysis of diabetic peripheral neuropathy in patients with Type 2 diabetes mellitus.

**Independent variables**	**OR (95% CI)**	**p** ** value**
Duration of diabetes	1.010 (1.004, 1.016)	**0.001**
Fasting plasma glucose	1.159 (1.017, 1.321)	**0.027**
Weight	1.042 (1.008, 1.077)	**0.016**
Waist circumference	1.046 (1.008, 1.086)	**0.019**
Visceral fat area	1.010 (1.000, 1.020)	**0.044**
Diabetic retinopathy	5.111 (2.081, 12.552)	**< 0.001**

*Note:* Values in bold indicate that < 0.05 is considered to possess statistical significance.

**Table 5:** Multivariate logistic regression analysis of diabetic peripheral neuropathy in patients with Type 2 diabetes mellitus.

**Independent variables**	**OR (95% CI)**	**p** ** value**
Duration of diabetes	1.009 (1.002, 1.016)	**0.007**
Glycated hemoglobin	1.120 (0.890, 1.410)	0.334
Fasting plasma glucose	1.115 (0.939, 1.323)	0.214
Uric acid	1.004 (0.999, 1.009)	0.100
Body mass index	0.811 (0.627, 1.049)	0.110
Visceral fat area	1.027 (1.004, 1.051)	**0.022**
Diabetic retinopathy	3.909 (1.373, 11.127)	**0.011**
Gender	2.351 (0.825, 6.702)	0.110

*Note:* Values in bold indicate that < 0.05 is considered to possess statistical significance.

## Data Availability

The data used to support the findings of this study have been included in this article.
